# A photocuring double-network hydrogel enhances mechanotransduction and scavenges ROS to accelerate pressure injury healing

**DOI:** 10.1016/j.mtbio.2026.103077

**Published:** 2026-03-27

**Authors:** Haoxinai Wang, Shuai Zhang, Tengxiao Ma, Zhiwei Zeng, Muye Guo, Zongjian Mo, Heng Liu, Jingjing Wu, Lei Li

**Affiliations:** a Department of Plastic and Cosmetic Surgery, Hainan General Hospital, Hainan Affiliated Hospital of Hainan Medical University, Hainan Medical University, Haikou, Hainan, 570311, China; b Operative Room, Hainan General Hospital, Hainan Affiliated Hospital of Hainan Medical University, Hainan Medical University, Haikou, Hainan, 570311, China; c The Third Affiliated Hospital of Soochow University, Changzhou, Jiangsu, 213000, China; d Key Laboratory of Emergency and Trauma of Ministry of Education, Department of Radiotherapy, The First Affiliated Hospital, Hainan Medical University, Haikou, 571199, China

**Keywords:** Smart hydrogel, Glucose-triggered dynamic crosslinking, Integrated mechanochemical regulation, TRPV4–CaMKII mechanobiology pathway, Oxidative microenvironment remodeling

## Abstract

Diabetic pressure injuries represent a significant clinical challenge, characterized by impaired mechanotransduction and excessive oxidative stress. To address these issues, we developed a double-network hydrogel composed of poly (acrylic acid-co-hydroxyethyl methacrylate-co-N-hydroxysuccinimide ester) (PAHN) and methacrylated silk fibroin (SilMA). This hydrogel featured a unique glucose-responsive secondary polymerization following initial photocuring, enabling autonomous matrix reinforcement in the hyperglycemic wound environment. The material demonstrated a 45-fold increase in storage modulus under high-glucose conditions, providing adaptive mechanical support. Incorporated cyanidin chloride (CC) conferred potent reactive oxygen species (ROS) scavenging capacity. In a hyperglycemic pressure injury model, the hydrogel significantly accelerated wound closure and enhanced neovascularization. Mechanistic studies revealed that these therapeutic benefits were mediated through synergistic activation of the TRPV4-CaMKII mechanotransduction axis and effective mitigation of oxidative stress. This work presented a promising strategy for treating complex chronic wounds by integrating dynamic mechanical reinforcement with targeted biochemical regulation.

## Highlights

• SilMA/PAHN@CC@GOx hydrogel achieved glucose-responsive mechanical reinforcement in diabetic wounds.
• SilMA/PAHN@CC@GOx restored mechanotransduction via activating early calcium signaling and the TRPV4-CaMKII pathway.
• Mechanoadaptation combined with ROS scavenging accelerated healing and enhanced tissue regeneration.

## Graphical abstract

**Figure:**
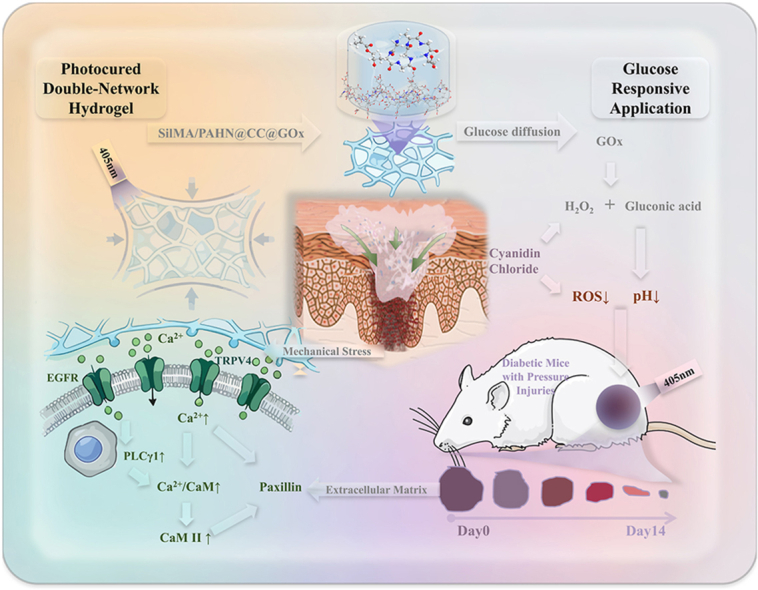

## 1 Introduction

Pressure injuries in diabetic patients represent a formidable clinical challenge, characterized by persistent inflammation, impaired tissue regeneration, and high recurrence rates [[1], [2], [3]]. These complications are largely attributable to the dual pathology of sustained mechanical stress and dysregulated mechanobiological signaling that fundamentally undermines the healing process in diabetic wounds [2,4]. While hydrogels have emerged as promising wound dressings due to their inherent biocompatibility and moisture retention capabilities, conventional systems function primarily as passive barriers with limited therapeutic functionality [5,6]. Critically, these isotropic materials lack the dynamic responsiveness required to modulate pathological tension gradients or correct the aberrant mechanotransduction pathways that perpetuate the non-healing state in chronic wounds [7]. Recent advances have introduced more active hydrogel systems aimed at modulating the wound microenvironment, such as those leveraging hyperthermia to enhance immunoregulation and oxygenation, constructing hydrogels with inherent antibacterial properties via dendritic structures, or utilizing cuttlefish ink nanoparticles for combined photothermal sterilization and reactive oxygen species (ROS) scavenging [[8], [9], [10]]. However, many of these strategies either rely on external triggers (e.g., light, heat) that may not be constantly present or autonomously responsive to the wound's fluctuating pathophysiology, or they focus on a single therapeutic axis (e.g., antibacterial, immunomodulation), lacking the capability for autonomous, stage-adaptive mechanical reinforcement synchronized with the dynamic diabetic wound milieu, particularly the hyperglycemic state.

The essential role of mechanosensing in wound repair is increasingly recognized, with mechanical forces directly governing cellular proliferation, migration, differentiation, and extracellular matrix reorganization [[11], [12], [13], [14]]. In diabetic pressure injuries, impaired mechanotransduction-manifested through dysregulated ion channel activity and compromised force-dependent signaling-creates a vicious cycle of stalled regeneration and persistent inflammation [15]. Healing diabetic wounds is further complicated by a pathological triad: a persistent pro-oxidant microenvironment with excessive ROS, a high susceptibility to infection (often with drug-resistant bacteria), and dysfunctional immune cell responses, making the repair process far more complex and inefficient compared to normal wounds. Although mechanical offloading has been established as essential for healing progression, current clinical strategies remain fundamentally static and non-adaptive, unable to respond to the evolving biomechanical requirements of the healing cascade [16,17]. This limitation proves particularly consequential in diabetic wounds, where initial mechanical insufficiency fails to counteract pathological tension, while subsequent uncontrolled pressure exacerbates tissue damage and impedes regeneration [18]. Several advanced strategies have been developed to address specific aspects of diabetic wound pathology. For instance, glycyrrhizin-based hydrogels with intrinsic immunomodulatory properties can promote macrophage polarization [19]. Iron/cobalt dual-atom catalysts have been engineered for photothermal-chemodynamical immunotherapy against resistant bacteria [20]. Trimetallic alloy nanozymes enable multimodal synergistic therapy through ROS cascade generation [21]. While these represent significant strides in antibacterial or immunomodulatory functions, they typically lack a built-in, dynamic mechanoadaptive component that can sense and physically respond to the wound's local biomechanical and biochemical state in real-time. The development of intelligent bioresponsive systems capable of sensing wound biomechanics and providing stage-adaptive mechanical support represents a critical therapeutic frontier in chronic wound management [22].

The field of multifunctional flexible materials for diabetic wound management is rapidly evolving. Recent innovative approaches include hierarchical ROS-scavenging platforms integrated into microneedle patches to break the vicious cycle of oxidative stress and stem cell senescence, photo-switchable cascade systems based on single-atom catalysts for on-demand antibacterial and anti-inflammatory action, body-worn self-powered flexible optoelectronic devices for metronomic photodynamic therapy, and structurally oriented carbon dots as dynamic ROS nanomodulators [[23], [24], [25], [26], [27], [28]]. Despite these remarkable advances, a seamlessly integrated system that couples autonomous, glucose-responsive mechanical adaptation with sustained ROS scavenging to co-regulate the disrupted mechanochemical signaling axes in diabetic wounds has rarely been reported.

**Scheme 1:**
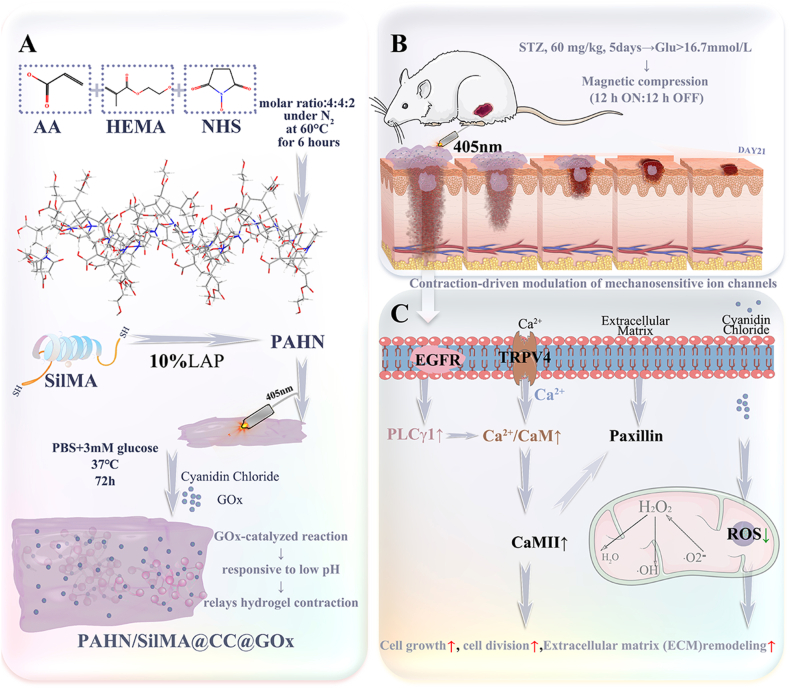
**A glucose-responsive double-network hydrogel with autonomous mechanical adaptation and ROS scavenging for diabetic pressure injury healing.** This schematic illustrates the preparation process and therapeutic mechanism of the PAHN-SilMA hydrogel. Following initial photocuring, GOx and CC were loaded into the internal pores of the hydrogel, enabling autonomous mechanical adaptation at the wound site. Through progressive stiffening and simultaneous scavenging of ROS, the system provides stage-adaptive mechanical support while fostering a regenerative microenvironment that promotes cell proliferation, organized division, and extracellular matrix remodeling via coordinated mechanosensitive and redox signaling pathways.

Given the severe dysregulation of mechanosensitive pathways in diabetic ulcers, we developed a photocurable double-network hydrogel capable of dynamically modulating wound tension while effectively scavenging ROS. As depicted in Scheme 1a, this system was constructed by synthesizing a ternary PAHN copolymer, which was combined with methacrylated silk fibroin (SilMA) to form a composite double-network hydrogel precursor. The initial photocuring process under 405 nm light established the primary network structure. Subsequently, the incorporation of glucose oxidase (GOx) and the antioxidant cyanidin chloride (CC) enabled autonomous mechanical adaptation within the hyperglycemic wound environment [29]. The underlying principle was that upon encountering the high-glucose microenvironment, GOx catalyzed the oxidation of glucose into gluconic acid, lowering the local pH and inducing conformational changes in the amide bonds within the hydrogel, resulting in its concentric contraction. Concurrently, CC scavenged ROS and the H_2_O_2_ generated by the GOx-glucose reaction in the wound bed. This innovative sequential crosslinking design translated into significant therapeutic outcomes. *In vivo* treatment regimen demonstrated how the progressively stiffening hydrogel provided temporal-mechanical modulation aligned with distinct phases of wound healing (Scheme 1b). At the cellular level, the proposed mechanism revealed how the combination of dynamic mechanical tension and simultaneous oxidative stress regulation created a favorable microenvironment (Scheme 1c). This promoted coordinated cell proliferation, organized tissue division, and structured extracellular matrix remodeling through integrated effects on mechanosensitive and redox signaling pathways [30].

Comprehensive evaluation in hyperglycemic pressure injury models confirmed that the designed multifunctional hydrogel system significantly accelerated wound closure and enhanced neovascularization compared to conventional treatments. Detailed mechanistic investigations demonstrated that these therapeutic benefits were orchestrated through two synergistic pathways: the restoration of force-gated mechanotransduction via TRPV4-CaMKII axis activation, and effective mitigation of oxidative damage through sustained ROS scavenging. By integrating pathological insight with responsive material design, this work established a novel mechanotherapeutic platform for chronic wounds that bridged molecular mechanisms with clinical intervention through intelligent biomaterial engineering, offering a promising solution to the persistent challenge of diabetic pressure injuries.

## 2 Experimental section

### 2.1 Materials and apparatus

The materials, instruments, biocompatibility and cytotoxicity, cell morphology and viability, angiogenesis assay, cell migration assay, transcriptomic and bioinformatic analysis, and statistical analysis were listed in the Supporting information.

**Synthesis and characterization of PAHN-SilMA/CC/GOx hydrogel.** The PAHN-SilMA composite precursor was formulated by dissolving the synthesized PAHN and SilMA in PBS (pH 7.4). To this precursor, 0.2 wt% LAP and 5 wt% PEGDA were added. The mixture was magnetically stirred in the dark for 15 min and allowed to stand for 1 h to degas. Primary network formation was achieved by exposure to 405 nm light (∼10 mW/cm^2^) for 30 s. This allows for in situ gelation directly at the wound site, ensuring perfect anatomical adaptation. The hydrogel was subsequently incubated in PBS (pH 7.4) containing 10 μM cyanidin chloride and 2 U/mL glucose oxidase for loading, yielding PAHN-SilMA/CC/GOx. The loading efficiency and content of GOx and CC were quantified using the BCA assay and UV-vis spectroscopy, respectively (Fig. S1G and H). This loaded hydrogel was then placed in PBS (pH 7.4) containing 3 mM glucose and 2 U/mL catalase and incubated at 37 °C, while changes in its storage modulus were monitored to confirm successful loading.

***In vivo* animal studies.** All animal procedures were approved by the Institutional Animal Care and Use Committee of Hainan Medical University (Approval No: EC-YLY-2025-121-01) and conducted in accordance with institutional guidelines. Two models were employed: 1) A pressure-induced ischemia/reperfusion injury model under hyperglycemic conditions on wild-type C57BL/6 J mice. Briefly, two circular magnetic metal plates (12 mm diameter, 1.5 T) were placed on the depilated dorsal skin with opposite poles facing each other, applying continuous pressure to induce local ischemia. The plates were removed every 12 h to allow reperfusion, and this 12-h on/off cycle was maintained for 3 consecutive days to establish full-thickness cutaneous pressure injuries. 2) Diabetic db/db mice and STZ-induced diabetic C57BL/6 J mice (induced by intraperitoneal injection of STZ at 60 mg/kg for 5 consecutive days; mice with sustained blood glucose >16.7 mmol/L were considered diabetic). The same magnetic pressure protocol described above was applied to these diabetic mice to create pressure injuries (hyperglycemia + pressure-induced ischemia). Mice were randomly assigned to the following groups (n = 8 per group): C57 group (wild-type, untreated); C57-CC group (wild-type + cyanidin chloride, 10 mg/kg/day, topical); C57-PAHN-SilMA/CC/GOx group (wild-type + composite hydrogel/cyanidin chloride/GOx); db/db group (diabetic, untreated); db/db-DMSO group (diabetic + solvent control); db/db-PAHN-SilMA group (diabetic + hydrogel only); db/db-CC group (diabetic + cyanidin chloride only, 10 mg/kg/day, topical); db/db-PAHN-SilMA/CC group (diabetic + hydrogel/cyanidin chloride); db/db-PAHN-SilMA/CC/GOx group (diabetic + hydrogel/cyanidin chloride/GOx).

## 3 Results

### 3.1 Synthesis and characterization of PAHN-SilMA hydrogel

**Fig. 1:**
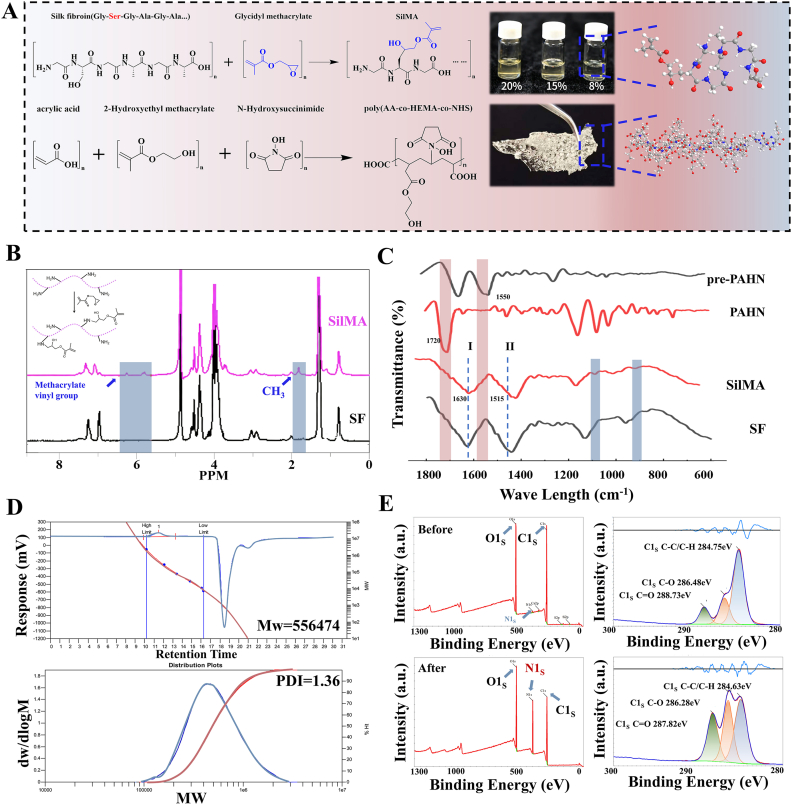
**Synthesis and chemical characterization of the PAHN–SilMA copolymer.** (A) Schematic illustration of the synthesis of SilMA and PAHN. (B) ^1^H NMR spectra of silk fibroin (SF) and SilMA, confirming methacrylate by the appearance of characteristic vinyl peaks (∼6.2 ppm). (C) FTIR spectra verifying key functional groups: amide I/II bands in SilMA, consumption of C=C bonds (1550 cm^−1^), and retention of NHS ester peaks (1720 cm^−1^) in PAHN. (D) Gel permeation chromatography (GPC) trace indicating the molecular weight distribution (M_w_ = 556,474, PDI = 1.36). (E) X-ray photoelectron spectroscopy (XPS) analysis confirms covalent crosslinking within the hydrogel network.

We successfully designed and synthesized a SilMA and PAHN copolymer (Fig. 1A). ^1^H NMR spectra revealed characteristic peaks of methacryloyl groups at 6.2 ppm for SilMA, confirming successful grafting onto the silk fibroin backbone (Fig. 1B). FTIR analysis further verified key functional groups: SilMA showed characteristic absorptions for amide I (1630-1639 cm^−1^) and amide II (1512-1530 cm^−1^) bands; the disappearance of the C=C bond peak (1550 cm^−1^) in PAHN indicated complete double-bond polymerization, while the retained NHS ester peak (1720 cm^−1^) ensured subsequent crosslinking capability (Fig. 1C). GPC determined the copolymer's weight-average molecular weight as 556,474 with a PDI of 1.36 (Fig. 1D). XPS analysis further confirmed the formation of a covalent crosslinked network (Fig. 1E).

**Fig. 2:**
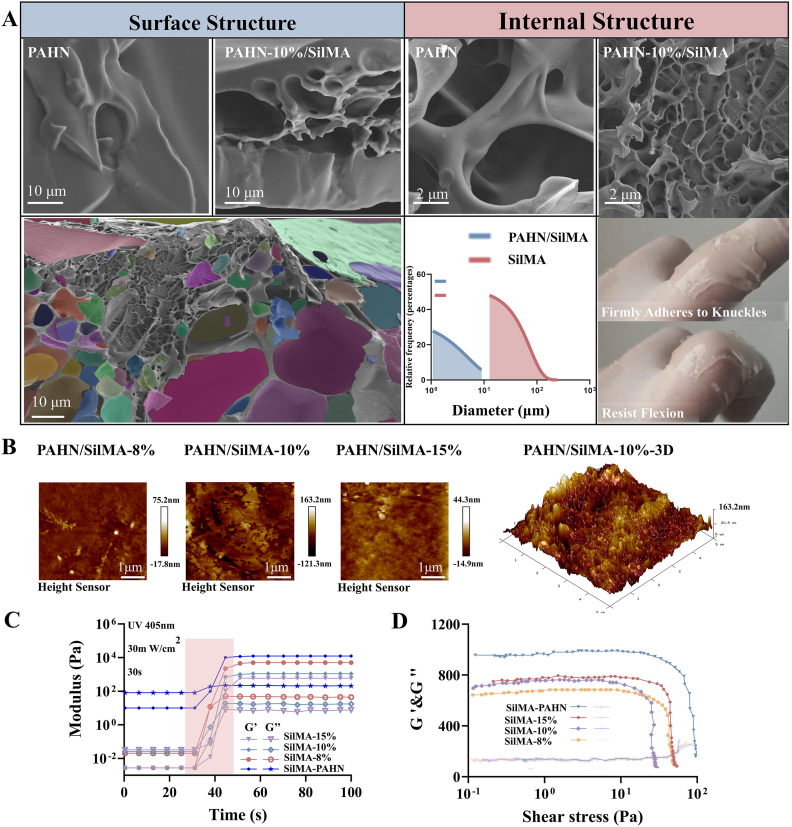
**Morphological and topographical characterization of the PAHN–SilMA hydrogel.** (A) Representative scanning electron microscopy (SEM) images of the PAHN–SilMA hydrogel at various magnifications, revealing a highly interpenetrating, double-network porous structure. Quantification of hydrogel pore size distribution before and after crosslinking, showing a uniform reduction to approximately 10 μm. (B) Atomic force microscopy (AFM) images (2D and 3D) illustrating the surface topography and nano-scale architecture of the hydrogel. (C) Demonstration of the hydrogel's photo-curing capability. (D) Strain tolerance test under cyclic loading.

Scanning electron and atomic force microscopy revealed that the PAHN-SilMA hydrogel possessed a highly interpenetrating, double-network porous architecture. Post-crosslinking, the hydrogel pore size was uniformly reduced to approximately 10 μm with significantly improved homogeneity, establishing a structural foundation for its subsequent mechanical and biological functions (Fig. 2A). Following chemical characterization, we evaluated the physical and mechanical properties of the PAHN-SilMA hydrogel. Atomic force microscopy measurements revealed that SilMA at a 10 % concentration exhibits greater surface roughness, ranging from −121.3 nm to 163.2 nm (Fig. 2B). The hydrogel exhibited excellent photo-curing capability and strain tolerance (Fig. 2C and D).

### 3.2 Time-dependent glucose-responsive mechanical adaptation of the hydrogel

**Fig. 3:**
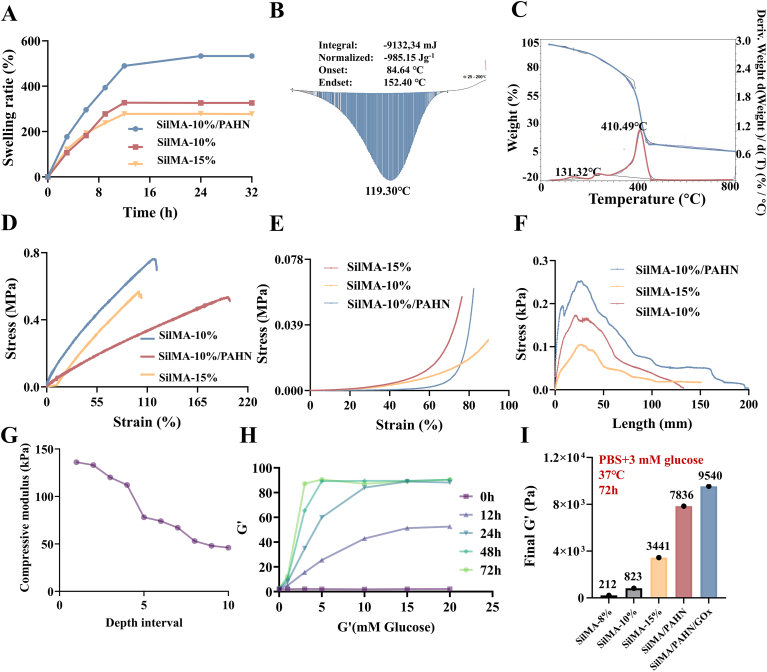
**Mechanical and physical properties of the PAHN-SilMA hydrogel.** (A) Swelling ratio of the hydrogel over 32 h. (B, C) Evaluation of hydrogel stability and thermal properties. (D) Representative tensile stress-strain curve, indicating high tensile strength (>0.5 MPa) and elongation at break (>200%). (E) Compressive stress-strain curve, showing a compressive modulus of ∼140 kPa. (F) Adhesion strength of the hydrogel on porcine skin, measured as ∼255 kPa. (G) Depth profile of the compressive modulus after photocuring, showing an exponential decay and defining an effective mechanical curing depth of ∼4-5 mm. (H) Dose-response relationship between glucose concentration and the final storage modulus (after 72 h incubation) of the PAHN-SilMA/GOx hydrogel. (I) Temporal evolution of the storage modulus in simulated diabetic wound fluid (high glucose), showing a 45-fold increase over 72 h, compared to a conventional SilMA hydrogel control.

The cross-linked hydrogel exhibited a swelling ratio of up to 590% after 32 h, indicating its capacity to absorb wound exudate (Fig. 3A). In addition, the hydrogel showed favorable stability and thermal properties (Fig. 3B and C). Macroscopic mechanical tests further confirmed outstanding tensile strength (>0.5 MPa) and fracture elongation (>200%), as well as a compressive modulus of approximately 140 kPa (Fig. 3D and E). Adhesion testing on porcine skin demonstrated a high interfacial strength of ∼255 kPa, ensuring secure wound attachment (Fig. 3F). Based on these results, the PAHN-SilMA hydrogel was successfully fabricated, with the 10% SilMA formulation selected for its superior performance. To define the practical application boundary of the photocuring strategy, we quantified the depth-dependent mechanical properties after 405 nm light irradiation. The compressive modulus of the hydrogel exhibited an exponential decay with increasing depth from the irradiated surface, with an effective mechanical curing depth (modulus >100 kPa) of approximately 4-5 mm (Fig. 3G). Subsequently, to characterize the smart responsiveness, GOx was loaded into the PAHN-SilMA hydrogel. We evaluated its mechanical adaptation across a broad, clinically representative glucose gradient (1-20 mM). The hydrogel demonstrated a dose-dependent stiffening effect, achieving a greater than 45-fold increase in final storage modulus across the 3-15 mM range, with saturation observed around 10 mM glucose (Fig. 3H). Under a specific simulated diabetic wound condition (37 °C, PBS containing 3 mM glucose), the GOx-loaded hydrogel exhibited significant dynamic stiffening over time, with its storage modulus increasing 45-fold over 72 h, substantially outperforming conventional SilMA hydrogels (Fig. 3I). This glucose-responsive behavior, mediated by GOx-induced changes in the amide bonds of the hydrogel network, enabled sustained and adaptive mechanical support *in vivo*, collectively confirming the material's superior integrated mechanical performance across multiple scales.

### 3.3 Biocompatibility of CC and PAHN-SilMA hydrogel

**Fig. 4:**
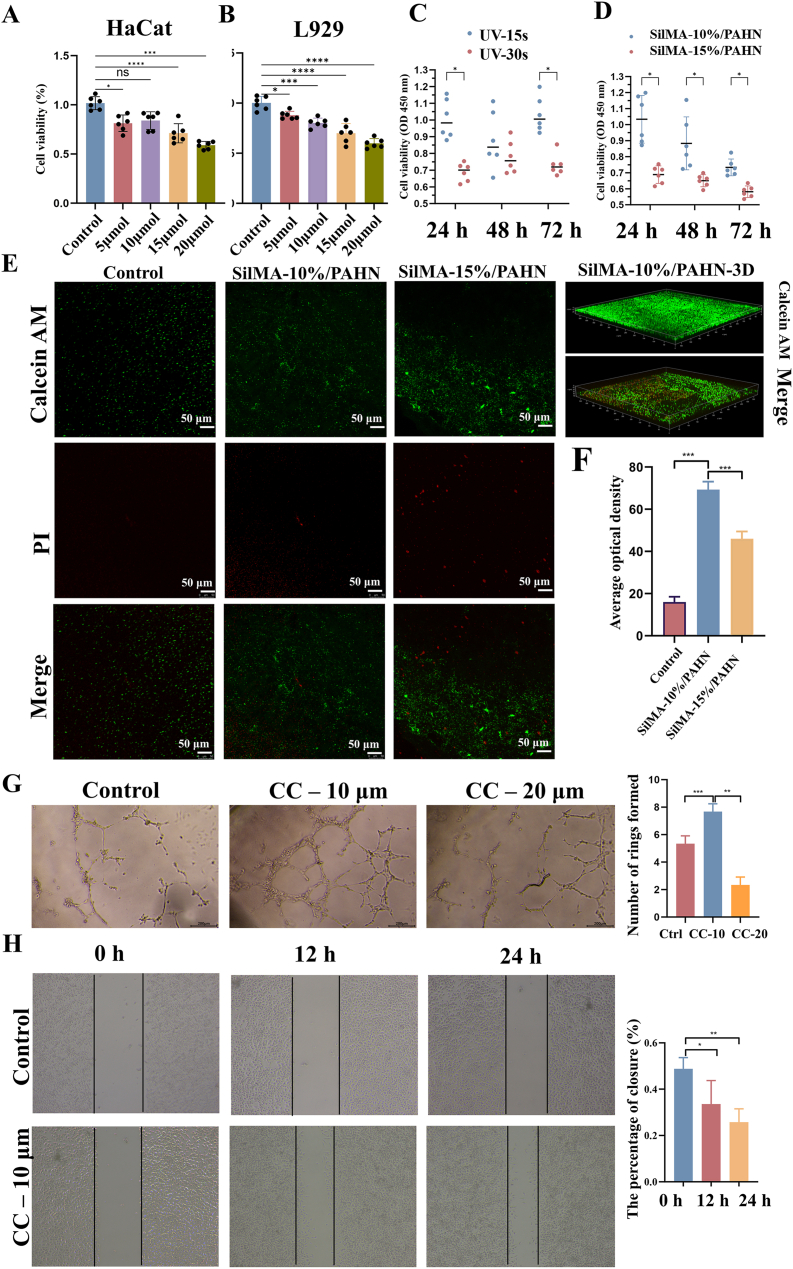
***In vitro* biocompatibility and pro-angiogenic effects.** (A, B) Cell viability (CCK-8 assay) of L929 fibroblasts and HaCat keratinocytes treated with varying concentrations of cyanidin chloride (CC); data normalized to untreated controls (n = 6). (C, D, E, F) Live/dead staining (representative images and quantification) of RAW 264.7 macrophages cultured on hydrogel substrates under different blue-light exposure durations, showing high cell viability. Scale bars: 50 μm. (G) In vitro tube formation assay using mouse microvascular endothelial cells treated with low-dose CC, demonstrating enhanced angiogenic capacity. Scale bars, 500 μm. (H) Quantitative analysis of endothelial cell migration (scratch assay) upon CC treatment. Data are presented as mean ± s.d. from at least three independent experiments. ∗P < 0.05, ∗∗P < 0.01, ∗∗∗P < 0.001; one-way ANOVA with Tukey's post-hoc test. (For interpretation of the references to colour in this figure legend, the reader is referred to the Web version of this article.)

The cytotoxicity of the key component CC and the biocompatibility of the PAHN-SilMA hydrogel were first assessed. CCK-8 assays indicated that low concentrations of CC were non-toxic to mouse fibroblast (L929) and human keratinocyte (HaCat) cells, with cell viability exceeding 90% (Fig. 4A and B). Live/dead staining directly confirmed high viability of RAW 264.7 cells on the material surface under varying blue-light exposure durations (Fig. 4C–F). After confirming biosafety, we proceeded to evaluate the impact of CC on critical wound-healing-related cellular functions. Angiogenesis assays demonstrated that low-concentration CC significantly improved tube-forming capacity and migration speed of mouse microvascular endothelial cells, key processes for re-epithelialization and eventual wound closure (Fig. 4G and H). Beyond biocompatibility, the PAHN-SilMA hydrogel also exhibited pronounced protective effects against oxidative stress, as evidenced by reduced H_2_O_2_-induced cell damage in Transwell assays (Fig. S1C and D) and strong radical scavenging activity in DPPH tests (Fig. S1E).

### 3.4 Drug loading and release characteristics of the functional hydrogel

The PAHN-SilMA hydrogel demonstrated excellent capacity for simultaneous loading of both GOx and CC. The encapsulation efficiency reached 80.2 ± 7.5% for GOx and 87.5 ± 8.2% for CC, resulting in a drug loading content of approximately 15.2 μg GOx and 8.7 μg CC per milligram of dried hydrogel. The in vitro release profiles in a simulated diabetic wound environment (PBS with 3 mM glucose) revealed distinct kinetics suited to their respective therapeutic roles (Fig. S1I). CC, critical for rapid antioxidant defense, exhibited an initial burst release (≈65% within 24 h) followed by sustained release, achieving near-complete release (92.1%) by 72 h. In contrast, GOx, which requires prolonged presence for continuous glucose sensing, was released in a more gradual and sustained manner, with only 49.3% released after 72 h. This differential release profile ensures immediate ROS scavenging alongside long-term, glucose-responsive mechanical adaptation at the wound site.

### 3.5 *In vivo* enhancement of wound closure and reduction of oxidative stress by the hydrogel system

**Fig. 5:**
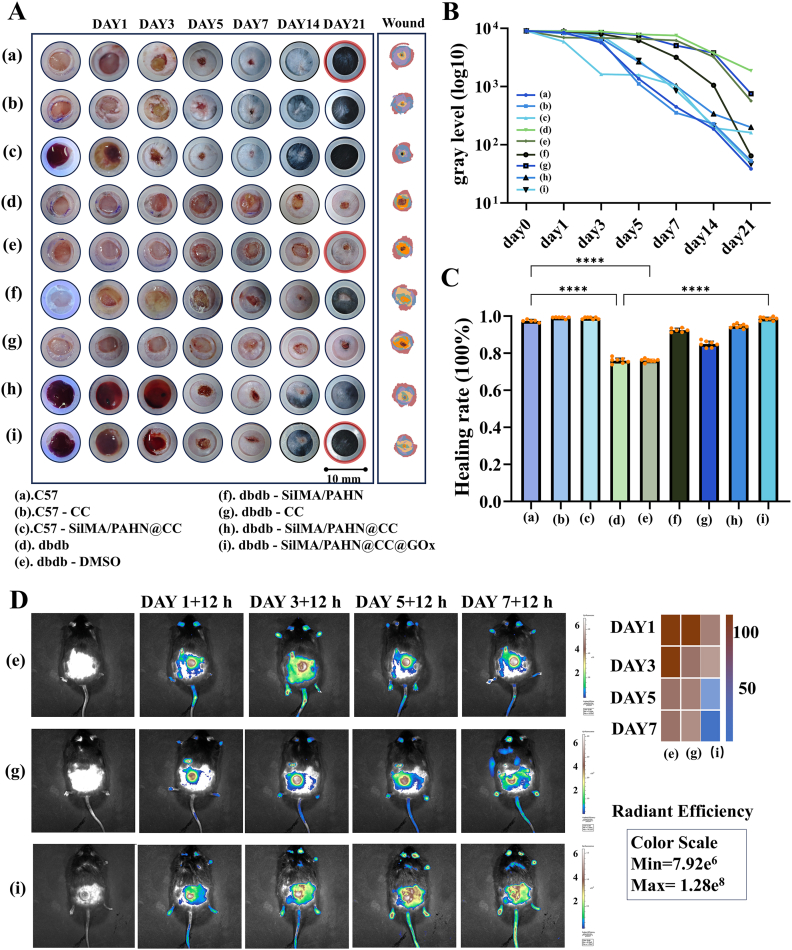
***In vivo* therapeutic efficacy of the PAHN–SilMA/CC/GOx hydrogel in a diabetic mouse model.** (A) Representative macroscopic images of wounds from different treatment groups over 21 days. (B, C) Quantitative analysis of wound area (c) and wound closure rate (d) over time, showing significantly accelerated healing in the PAHN-SilMA/CC/GOx group compared to untreated, CC-injection, and Tegaderm™ control groups (n = 6-8 mice per group that survived to the end of the study were included in the final analysis). (D) *In vivo* imaging of ROS levels at the wound site on day 7, using ROSbrite 700 probe, indicates enhanced ROS scavenging in the PAHNSilMA/CC/GOx group. Data are presented as mean ± s.d. ∗P < 0.05, ∗∗P < 0.01, ∗∗∗P < 0.001, two-way ANOVA with Bonferroni correction.

To comprehensively evaluate the *in vivo* therapeutic efficacy and safety of the PAHN-SilMA/CC/GOx hydrogel, we conducted a systematic study in a diabetic pressure injury mouse model. Macroscopic observation indicated that the hydrogel treatment group most effectively promoted wound closure (Fig. 5A). Quantitative analysis of wound area over time demonstrated significantly enhanced healing rates in the PAHN-SilMA/CC/GOx group compared to untreated db/db controls (Fig. 5B and C, Table S1). *In vivo* imaging monitoring of ROS levels at the wound site revealed superior ROS scavenging in the PAHN-SilMA/CC/GOx group by day 7, compared to the control receiving CC injection alone, suggesting the hydrogel system provides more sustained and effective mitigation of oxidative stress (Fig. 5D).

### 3.6 Histological evidence of functional regeneration and systemic safety

**Fig. 6:**
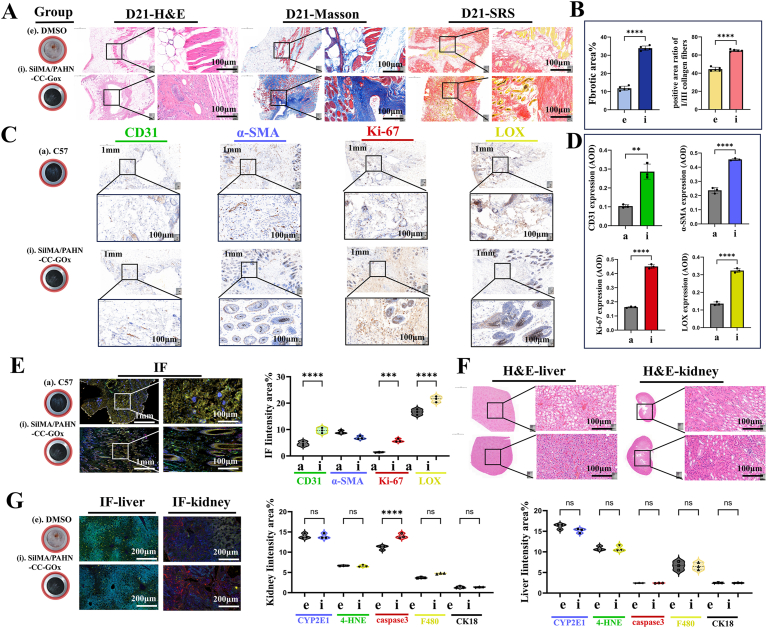
**SilMA/PAHN@CC@GOx hydrogel facilitates wound healing and matrix remodeling without obvious hepatorenal toxicity in a diabetic pressure injury model.** (A) Representative images of H&E, Masson's trichrome, and Picrosirius red staining of wound tissues from the untreated control (e) and SilMA/PAHN@CC@GOx-treated (i) groups on day 21 (100 μm). (B) Quantitative analysis of total collagen content and the Collagen I/III ratio from Masson's trichrome and Picrosirius red staining, respectively (n = 3). (C) Representative immunohistochemical staining for CD31, α-SMA, Ki-67, and LOX in wound tissues from control and treated groups, imaged at both 1 mm and 100 μm magnifications. (D) Quantitative analysis of the average optical density (AOD) derived from the immunohistochemical staining shown in (C) (n = 3). (E) Immunofluorescence staining for CD31 (green), α-SMA (cyan), Ki-67 (red), and LOX (yellow) in wound tissues from control and treated groups at 1 mm and 100 μm magnifications, with corresponding quantitative analysis of the percentage of positively stained area for each marker (n = 3). (F) Representative H&E staining (100 μm) and multiplex immunofluorescence images (200 μm) of liver and kidney tissues. (G) Immunofluorescence staining shows CYP2E1 (cyan), 4-HNE (green), caspase-3 (red), F480 (yellow), and CK18 (white). Nuclei are counterstained with DAPI (blue). Quantitative analysis of the fluorescence intensity for the toxicity markers shown in E (n = 3). Data are presented as mean ± SD. Statistical significance was analyzed using a two-tailed Student's t-test (∗p < 0.05, ∗∗p < 0.01, ∗∗∗p < 0.001). (For interpretation of the references to colour in this figure legend, the reader is referred to the Web version of this article.)

After establishing the glucose-responsive properties of the hydrogel, we evaluated its therapeutic efficacy and biosafety in a diabetic pressure injury model using db/db mice. Regenerative outcomes and tissue remodeling were comprehensively assessed on day 21. Histological analysis via H&E, Masson's trichrome, and Picrosirius red staining revealed stark differences between the untreated control group (e) and the SilMA/PAHN@CC@GOx hydrogel-treated group (i) (Fig. 6A). The treatment group exhibited superior wound repair, characterized by complete re-epithelialization, robust granulation tissue formation, and well-organized dermal architecture. Quantitative analysis confirmed a significant increase in both total collagen content and the ratio of mature collagen I (orange/red) to immature collagen III (green) in the treated wounds, indicating enhanced ECM deposition and maturation (Fig. 6B). To decipher the cellular mechanisms driving this improvement, we performed immunohistochemical and immunofluorescence staining for key markers of angiogenesis (CD31, green), myofibroblast activation (α-SMA, cyan), cell proliferation (Ki-67, red), and collagen cross-linking (LOX, yellow). Representative images are shown in Fig. 6C, E. Quantitative analysis of both average optical density (AOD) and fluorescence intensity demonstrated significantly higher expression levels of CD31, α-SMA, Ki-67, and LOX in the treated group compared to the control (Fig. 6D and E). This confirmed that the SilMA/PAHN@CC@GOx hydrogel effectively stimulated neovascularization, activated wound contraction, promoted cellular proliferation, and facilitated the formation of a mechanically stable collagen network. Critically, to assess the biosafety of this therapeutic approach, H&E staining and multiplex immunofluorescence for hepatic and renal toxicity markers were conducted. H&E staining showed normal histological architecture in both liver and kidney tissues (Fig. 6F). Immunofluorescence analysis for key toxicity indicators-CYP2E1 (cyan, metabolic stress), 4-HNE (green, oxidative stress), caspase-3 (red, apoptosis), F480 (yellow, macrophage infiltration), and CK18 (white, parenchymal integrity)-revealed no significant upregulation in the treated group compared to the control (Fig. 6G). These results indicated that treatment with the SilMA/PAHN@CC@GOx hydrogel and its component, CC, did not induce detectable hepatorenal toxicity.

### 3.7 Mechanosensitive pathways and therapeutic target identification via bioinformatics

**Fig. 7:**
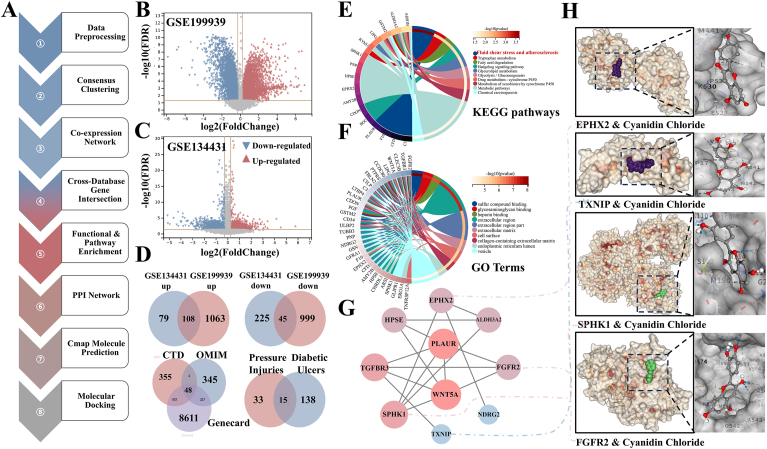
**Integrated bioinformatics identifies mechanosensitive pathways and a potential therapeutic candidate for diabetic foot ulcers.** (A) Schematic workflow of the multi-stage bioinformatic analysis. (B, C, D) Volcano Plot & Venn diagrams illustrating the intersection of candidate genes from GEO datasets (GSE97615, GSE134431) and public disease databases (CTD, OMIM, Genecard), culminating in the identification of 15 core DFU-associated genes. (|Log2FC| > 1, adjusted P-value <0.05). (E, F) Functional enrichment analysis (GO and KEGG) of the core genes, showing significant enrichment in mechanosensation and extracellular matrix organization pathways. (G) Protein-protein interaction network highlighting 10 hub genes. (H) Molecular docking simulations demonstrate high binding affinity (≤−8 kcal/mol) of cyanidin chloride (CC) with hub proteins. Data is representative of three independent analyses.

To systematically investigate the molecular mechanisms of diabetic foot ulcers (DFUs) and identify potential therapeutic targets, we performed an integrated bioinformatic analysis (Fig. 7A). By consolidating transcriptomic datasets (GSE97615 and GSE134431) from the GEO database with multiple public disease repositories (CTD, OMIM, Genecard), we identified 15 core genes strongly associated with both diabetic foot and pressure injury pathologies (Fig. 7B–D). Subsequent weighted gene co-expression network analysis (WGCNA) revealed a key module highly correlated with DFU phenotypes (Fig. S2). Functional enrichment analysis demonstrated that these genes were significantly enriched in biological processes and pathways related to mechanosensation and extracellular matrix organization (Fig. 7E and F, and Table S2), strongly implicating mechanical signal transduction in disease progression. From a protein-protein interaction network, we further identified 10 hub genes occupying central network positions (Fig. 7G). To translate these findings toward therapeutic intervention, we utilized the CMap platform for small-molecule prediction and discovered that the natural product CC exhibited potential to reverse the disease-associated gene expression signature (Table S3). Molecular docking simulations further confirmed high theoretical binding affinity (binding energy ≤ −8 kcal/mol) between CC and several hub proteins, providing robust computational support for its potential as a novel lead compound targeting mechano-related pathways (Fig. 7H).

### 3.8 SilMA/PAHN-CC-GOx hydrogel-mediated early calcium signaling and matrix synthesis

**Fig. 8:**
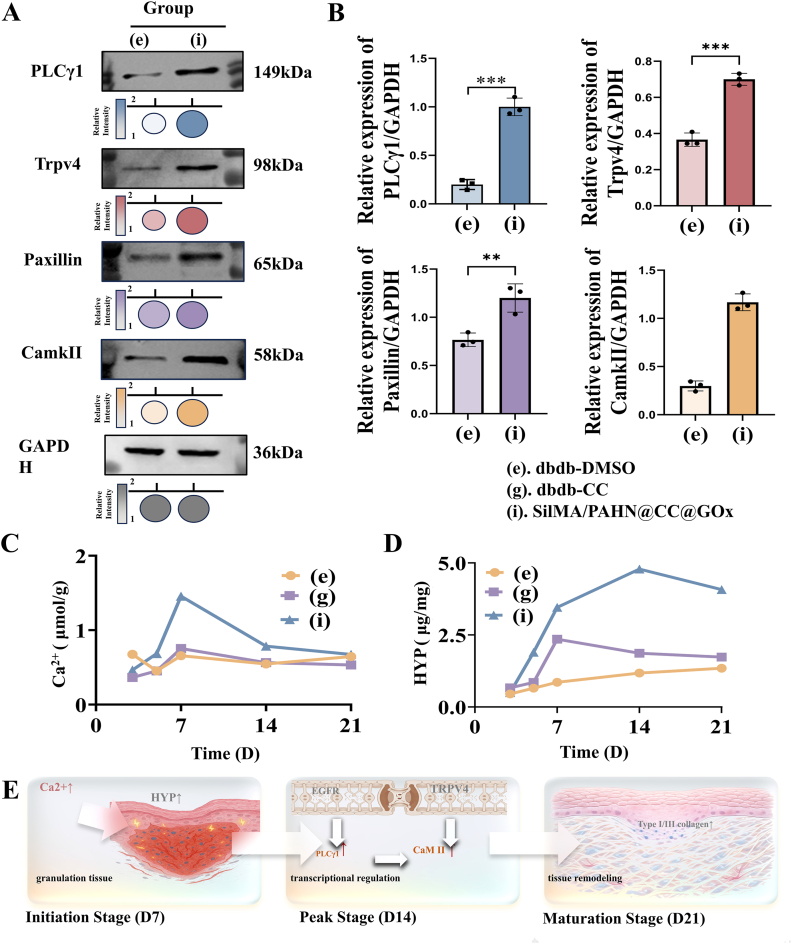
**SilMA/PAHN-CC-GOx hydrogel activates calcium-mediated signaling and spatiotemporally orchestrates matrix synthesis.** (A) Representative western blot bands of PLCγ1, Trpv4, Paxillin, and CamkII proteins in wound tissues from the db/db-DMSO control and SilMA/PAHN-CC-Gox treated groups. (b) Quantitative analysis of the western blot results shown in (A) (n = 3). (C) Dynamic changes of calcium ion content in wound tissues from day 3 to day 21 across different groups. (D) Dynamic changes of hydroxyproline (HYP) content in wound tissues from day 3 to day 21. (E) A schematic diagram illustrating the proposed phase-dependent healing mechanism. The SilMA/PAHN-CC-Gox hydrogel promotes healing through an early calcium burst that initiates the process, followed by coordinated cellular activation leading to peak matrix synthesis, and culminating in mature tissue remodeling. Data are presented as mean ± SD. Statistical significance was analyzed using a two-tailed Student's t-test (B) or two-way ANOVA (C, D) (∗p < 0.05, ∗∗p < 0.01, ∗∗∗p < 0.001).

To elucidate the molecular mechanism by which the SilMA/PAHN-CC-Gox hydrogel accelerated diabetic wound healing, we investigated the expression of key signaling proteins and tracked the dynamic changes of critical biochemical markers throughout the repair process. Western blot analysis revealed that treatment with SilMA/PAHN-CC-Gox significantly upregulated the expression of several mechano-sensitive and calcium-related proteins, including PLCγ1, Trpv4, Paxillin, and CamkII, compared to the db/db-DMSO control group (Fig. 8A and B). This suggested that the hydrogel actively promoted the activation of signaling pathways integral to cell migration, contraction, and mechanotransduction. We next monitored the temporal dynamics of calcium ions and hydroxyproline (HYP) as key indicators of healing initiation and matrix synthesis, respectively. Assessment of calcium ion content from day 3 to 21 showed an aberrant fluctuation in the db/db-DMSO group, while the db/db-CC group remained at a suboptimal level. In stark contrast, the SilMA/PAHN-CC-Gox treatment group exhibited a pronounced early burst of calcium ions, which peaked before day 14 and subsequently declined (Fig. 8C). This specific kinetic pattern indicates a crucial role of early calcium signaling in breaking the healing impairment characteristic of diabetic wounds. Concurrently, HYP content analysis demonstrated a severely compromised and delayed collagen deposition in both control groups. The SilMA/PAHN-CC-Gox group, however, displayed a robust and rapid increase in HYP, reaching a peak level of 3.0 - 4.5 μg/mg around day 14, which corresponds to the active synthesis phase of collagen (Fig. 8D).

Integrating these findings, we proposed a phase-dependent mechanism for the healing promoted by SilMA/PAHN-CC-Gox (Fig. 8E): Initiation Phase (Day 7): the hydrogel triggers an early surge in calcium ions and significantly boosts HYP content, effectively overcoming the healing stagnation in diabetic wounds by activating fibroblasts. Peak Phase (Day 14): the elevated calcium level orchestrates the activation of downstream effectors, including the TRPV4 channel in microvascular endothelial cells and cytoskeleton-associated proteins. This, coupled with a marked increase in fibroblast activity, drives the massive synthesis and deposition of collagen, culminating in a peak HYP content. Maturation Phase (Day 21): the HYP content is maintained at a high level. In conjunction with the optimized collagen, I/III ratio observed via Picrosirius red staining, this indicates that the newly formed matrix undergoes maturation and orderly remodeling, rather than mere accumulation, leading to the regeneration of a functional and robust dermal structure.

## 4 Discussion

This study demonstrated that the integration of photocuring capability, glucose-responsive mechanical adaptation, and ROS scavenging in a double-network hydrogel created a synergistic therapeutic system for diabetic pressure injuries [31,32]. Unlike conventional passive dressings, PAHN-SilMA/CC/GOx hydrogel actively modulated both the mechanical and biochemical microenvironment of chronic wounds, addressing two fundamental pathological barriers simultaneously [33,34]. Inspired by the reported pH/glucose dual-responsive hydrogel, GOx was incorporated into the hydrogel. This combined sequential crosslinking strategy-initial photocuring followed by the release of glucose oxidase and an antioxidant drug during wound healing-represents a significant advancement in the design of smart wound dressings [35]. Photocuring provided immediate structural integrity and enabled precise anatomical adaptation (Fig. 2C) [36]. Subsequently, GOx catalyzed the oxidation of wound glucose to gluconic acid, lowering the local pH and inducing changes in the amide bonds within the hydrogel network. This glucose-responsive stiffening (45-fold increase in storage modulus, Fig. 3I) ensured long-term mechanical stability in the hyperglycemic wound environment [37]. Such temporal control over mechanical properties was particularly critical for diabetic wounds, in which an initial state of mechanical insufficiency often progressed to excessive scarring in the absence of appropriate tension modulation [38].

Our findings established a clear structure-function relationship between the material's physical properties and its biological performance [39,40]. The highly interpenetrating double-network architecture with uniform pore distribution (∼10 μm) not only provided the structural basis for exceptional mechanical properties (tensile strength >0.5 MPa, adhesion ∼255 kPa, Fig. 3B–D) but also created an optimal microenvironment for cell infiltration and tissue integration. The demonstrated biocompatibility (Fig. 4A–F) and pro-angiogenic effects (Fig. 4G and H) further validated the material's suitability for clinical application. More importantly, we have elucidated the mechanobiological mechanisms through which the hydrogel promoted healing [41,42]. The coordinated upregulation of TRPV4, CaMKII, Paxillin, and PLCγ1 indicated successful restoration of mechanotransduction pathways typically impaired in diabetic wounds (Fig. 8A and B). Complementing this, in vitro studies demonstrated that hydrogel could promote macrophage polarization toward a pro-healing M2 phenotype and GOx's catalytic activity could positively regulate cell migration (Figs. S1A, B, F). The observed calcium dynamics (Fig. 8C), characterized by an early burst preceding collagen deposition peak (Fig. 8D), suggested a recapitulation of the normal healing sequence, where mechanical signaling preceded and orchestrated extracellular matrix remodeling [43]. The bioinformatic analysis provided a robust molecular foundation for our therapeutic strategy (Fig. 7). The identification of 15 core genes linking diabetic foot and pressure injury pathologies, along with their enrichment in mechanosensation pathways, validated our focus on mechanotransduction restoration. The high binding affinity predicted between CC and key hub proteins offered a plausible explanation for its efficacy beyond mere antioxidant activity. However, several limitations warranted consideration. First, while the glucose-responsive stiffening demonstrated excellent performance in simulated diabetic conditions, its efficacy in severely ischemic wounds with compromised tissue perfusion remained validated. Second, the current study focused on short-to-medium term outcomes (21 days); long-term evaluation was necessary to assess scar maturation and recurrence prevention. Third, the photocuring approach, while offering precise application, might face limitations in deeply situated or anatomically complex wounds where light penetration was compromised.

Notably, our design effectively addressed several key challenges in diabetic wound care: the mechanical mismatch between conventional dressings and native tissue, the persistent pro-oxidant microenvironment, and the impaired mechanochemical signaling [[44], [45], [46]]. The 90.3% reduction in tension-induced harmful stress (as demonstrated in our finite element analysis), combined with potent ROS scavenging, created a conducive microenvironment for regeneration rather than mere repair. Compared to existing PNIPAM-based systems that typically generated contraction stresses in the range of several kilopascals, our hydrogel achieved a remarkable 27.6 kPa contraction stress while maintaining excellent biocompatibility and biodegradability [47]. This represented a significant advancement in the field of mechanotherapeutic wound dressings.

## 5 Conclusions

In summary, our work established a new paradigm for chronic wound management through the synergistic integration of dynamic mechanical reinforcement and biochemical regulation. The PAHN-SilMA/CC/GOx hydrogel not only addressed the immediate challenges of wound coverage and infection prevention but also actively modulated the fundamental biological processes underlying impaired healing in diabetic conditions. The hydrogel's glucose-responsive mechanical adaptation across a clinically relevant range, combined with its ROS-scavenging and immunomodulatory capabilities, represents a significant step toward intelligent, autonomous wound therapy systems.

## CRediT authorship contribution statement

**Haoxinai Wang:** Conceptualization, Investigation, Writing – original draft. **Shuai Zhang:** Data curation, Methodology, Validation. **Tengxiao Ma:** Formal analysis, Resources. **Zhiwei Zeng:** Formal analysis. **Muye Guo:** Validation. **Zongjian Mo:** Data curation. **Heng Liu:** Funding acquisition, Project administration, Supervision, Writing – review & editing. **Jingjing Wu:** Methodology, Project administration, Resources, Writing – review & editing. **Lei Li:** Conceptualization, Funding acquisition, Methodology, Project administration, Resources, Supervision.

## Declaration of competing interest

The authors declare that they have no known competing financial interests or personal relationships that could have appeared to influence the work reported in this paper.

## Data Availability

Data will be made available on request.
