# Supplementary material for: A photocuring double-network hydrogel enhances mechanotransduction and scavenges ROS to accelerate pressure injury healing
Source: Mater Today Bio. 2026 Mar 27;38:103077. doi: 10.1016/j.mtbio.2026.103077 (PMC13087789; doi:10.1016/j.mtbio.2026.103077)
Supplement: Multimedia component 1 [file mmc1.docx]

**Supporting Information**

**A photocuring double-network hydrogel enhances mechanotransduction and scavenges ROS to accelerate pressure injury healing**

Haoxinai Wang^a,#^, Shuai Zhang^b,#^, Tengxiao Ma^a^, Zhiwei Zeng^a^, Muye Guo^a^, Zongjian Mo^c^, Heng Liu^a,d,^*, Jingjing Wu^a,^*, Lei Li^a,^*

^a^ Department of Plastic and Cosmetic Surgery, Hainan General Hospital, Hainan Affiliated Hospital of Hainan Medical University, Hainan Medical University, Haikou, Hainan, 570311, China

^b^ Operative room, Hainan General Hospital, Hainan Affiliated Hospital of Hainan Medical University, Hainan Medical University, Haikou, Hainan, 570311, China

^c^ The Third Affiliated Hospital of Soochow University, Changzhou, Jiangsu, 213000, China

^d^ Key Laboratory of Emergency and Trauma of Ministry of Education, Department of Radiotherapy, The First Affiliated Hospital, Hainan Medical University, Haikou 571199, China

To whom correspondence should be addressed:

E-mail: [liuheng11b@muhn.edu.cn](mailto:liuheng11b@muhn.edu.cn) (H. Liu); [wujingjing57714@muhn.edu.cn](mailto:wujingjing57714@muhn.edu.cn) (J.J. Wu); [hy303913@muhn.edu.cn](mailto:hy303913@muhn.edu.cn) (L. Li)

^#^These two authors contributed equally to this work (H. X. A. Wang and S. Zhang).

**Table of contents**

1. General experimental section
2. Additional characterization and mechanistic studies of the PAHN‑SilMA hydrogel system.
3. Bioinformatics analysis workflow and functional profiling of transcriptomic data from diabetic foot ulcers
4. Statistics on the dynamic changes in wound closure rate
5. Information on enriched KEGG pathways
6. Cyanidin chloride reverses disease-associated gene expression signature

**1. General Experimental Section**

**Materials.** Acrylic acid (AA), 2-hydroxyethyl methacrylate (HEMA), N-hydroxysuccinimide ester (NHS-ester), ammonium persulfate (APS), poly (ethylene glycol) diacrylate (PEGDA, Mn = 700), glucose oxidase (GOx, ≥100 U/mg), iron (II) chloride tetrahydrate (FeCl_2_·4H_2_O), and cyanidin chloride were purchased from Sigma-Aldrich (St. Louis, MO, USA). Lithium Phenyl (2,4,6-trimethylbenzoyl) phosphinate (LAP) was obtained from TCI Chemicals. Methacrylated silk fibroin (SilMA) was synthesized in-house as previously described. All cell culture reagents, including the Cell Counting Kit-8 (CCK-8) and Calcein-AM/PI Live/Dead Stain Kit, were sourced from Wuhan Servicbio Biotechnology Co., Ltd. (China). Calcium and hydroxyproline assay kits were purchased from Beyotime Biotechnology (China). Human keratinocytes (HaCaT), mouse fibroblasts (L929), RAW 264.7 macrophages, and mouse dermal microvascular endothelial cells (CP-M201) were procured from Procell Life Science & Technology Co., Ltd. (China). Primary antibodies against α-SMA (ab7817), CD31 (ab28364), Ki-67 (ab15580), LOX (ab174316), TRPV4 (ab39260), Paxillin (ab32084), PLCγ1 (ab76155), CaMKII (ab52476), and corresponding secondary antibodies were acquired from Abcam (Cambridge, UK).

**Synthesis and characterization of PAHN-SilMA hydrogel**

**Synthesis of PAHN terpolymer.** The terpolymer PAHN was synthesized via free radical polymerization. Monomers AA, HEMA, and NHS-ester at a molar ratio of 4:4:2 was dissolved in deionized water under a nitrogen atmosphere. Polymerization was initiated with 0.1 wt% APS and proceeded at 60℃ for 6 h. The resultant product was dialyzed (MWCO 3500 Da) against deionized water for 72 h, with water changes every 12 h, followed by lyophilization.

**Hydrogel fabrication.** The PAHN-SilMA composite precursor was formulated by dissolving the synthesized PAHN and SilMA in PBS (pH 7.4). To this precursor, 0.2 wt% LAP and 5 wt% PEGDA were added. The mixture was magnetically stirred in the dark for 15 min and allowed to stand for 1 h to degas. Primary network formation was achieved by exposure to 405 nm light (~10 mW/cm^2^) for 30 s. The hydrogel was subsequently incubated in PBS (pH 7.4) containing 10 μM cyanidin chloride and 2 U/mL glucose oxidase for loading, yielding PAHN‑SilMA/CC/GOx. This loaded hydrogel was then placed in PBS (pH 7.4) containing 3 mM glucose and 2 U/mL catalase and incubated at 37 ℃, while changes in its storage modulus were monitored to confirm successful loading.

**Hydrogel characterization.** The chemical structure of PAHN and the composite hydrogel was verified by ^1^H NMR (Bruker Avance III HD, 400 MHz) and FTIR spectroscopy (Nicolet iS50). Rheological properties (storage modulus G' and loss modulus G'') were measured using a rotational rheometer (Anton Paar MCR 302) at 1% strain and 1 Hz frequency. Compressive modulus was determined via uniaxial compression testing (Instron 5943) at a strain rate of 2 mm/min. Micro-morphology was observed by scanning electron microscopy (SEM, Hitachi SU8010) at 5 kV. The equilibrium swelling ratio and in vitro degradation profile in PBS (pH 7.4, 37°C) were determined gravimetrically. All characterization experiments were performed with at least three independent samples (n≥3).

***In vitro* cellular assays.** All cell cultures were maintained under standard conditions, and experiments were conducted with a minimum of three biological replicates (n=3).

**Biocompatibility and cytotoxicity.** The cytotoxicity of hydrogel extracts towards HaCaT keratinocytes and L929 fibroblasts was assessed using the CCK-8 assay after 1, 3, and 5 days of culture. Cells cultured in standard medium served as the control.

**Cell morphology and viability.** Live/Dead staining (Calcein-AM/PI) and cell morphology of HaCaT, L929, and RAW 264.7 macrophages seeded on hydrogel surfaces were visualized using confocal laser scanning microscopy (Leica TCS SP8).

**Angiogenesis assay.** The tube formation capability of CP-M201 endothelial cells was evaluated using a Matrigel assay following treatment with Cyanidin Chloride (0, 10, 20 µM) for 6 h. Tube length and branch points were quantified from three random fields per well using ImageJ software.

**Cell migration assay:** A scratch wound assay was performed on L929 monolayers. Cell migration into the scratch area was monitored and quantified at 0, 12, and 24 h after wound creation and the addition of serum-free medium containing 10 µM Cyanidin Chloride.

**Treatment and evaluation.**

**Wound closure.** The wound area was photographed periodically, and the closure rate was calculated using ImageJ by two independent blinded researchers.

**Histological analysis.** At days 7 and 14 post-treatment, wound and organ tissues were harvested, fixed, paraffin-embedded, and sectioned. Histological evaluation was performed using H&E, Masson's trichrome, and Sirius red staining. Immunofluorescence was conducted for markers in wound (α-SMA, CD31, Ki-67, LOX), liver (CYP2E1, 4-HNE, Caspase-3, F4/80, CK18), and kidney (Caspase-3, Megalin, WT1, CD31). Analysis was performed on sections from three different tissue regions per sample by blinded assessors.

**Biochemical assays.** Calcium (Ca²⁺) and hydroxyproline content in wound tissues at days 3, 5, 7, 14, and 21 were quantified using commercial assay kits (Beyotime, China) according to the manufacturer's instructions.

**Oxidative stress assessment.** In vivo ROS levels at the wound site were detected 24 h post-treatment using the ROSBrite 700 fluorescence probe (5 mg/kg, intravenous) and an *in vivo* imaging system (IVIS, PerkinElmer). Fluorescence intensity was quantified using Living Image software.

**Western blot analysis.** Protein expression of TRPV4, Paxillin, CaMKII, and PLCγ1 in wound tissues was analyzed by Western blot. Total protein was extracted, separated by SDS-PAGE, transferred to PVDF membranes, and probed with specific primary antibodies followed by HRP-conjugated secondary antibodies. Band intensities were quantified using ImageJ software.

**Transcriptomic and bioinformatic analysis**

Datasets GSE97615 and GSE134431 were downloaded from the GEO database. Data preprocessing, normalization, and batch effect removal were performed using R. Differential expression analysis between diabetic foot ulcer and control tissues was conducted using *edgeR* and *DESeq2* (|Log2FC| > 1, adjusted P-value < 0.05). Weighted Gene Co-expression Network Analysis (WGCNA) was employed to identify disease-associated modules. Pressure injury-related gene lists from CTD, OMIM, and Genecards were intersected with DEGs to identify candidate genes. Functional enrichment analysis (GO and KEGG) was performed using *clusterProfiler*. A Protein-Protein Interaction (PPI) network was constructed using the STRING database, and hub genes were identified with CytoHubba. The CMap database was queried to predict potential therapeutics. Molecular docking between Cyanidin Chloride and the top 10 hub gene-encoded proteins was performed using AutoDock Vina.

**Statistical analysis**

All quantitative data are presented as mean ± standard deviation (SD). Statistical analysis was performed using SPSS (version 26.0) and R (version 4.2.1). For comparisons between two groups, a two-tailed Student’s t-test (for normally distributed data) or Mann-Whitney U test (for non-normally distributed data) was used. For multiple group comparisons, one-way or two-way analysis of variance (ANOVA) was applied, followed by Tukey's or Sidak's post-hoc test, respectively. A p-value < 0.05 was considered statistically significant.

**2. Additional characterization and mechanistic studies of the PAHN‑SilMA hydrogel system**

**
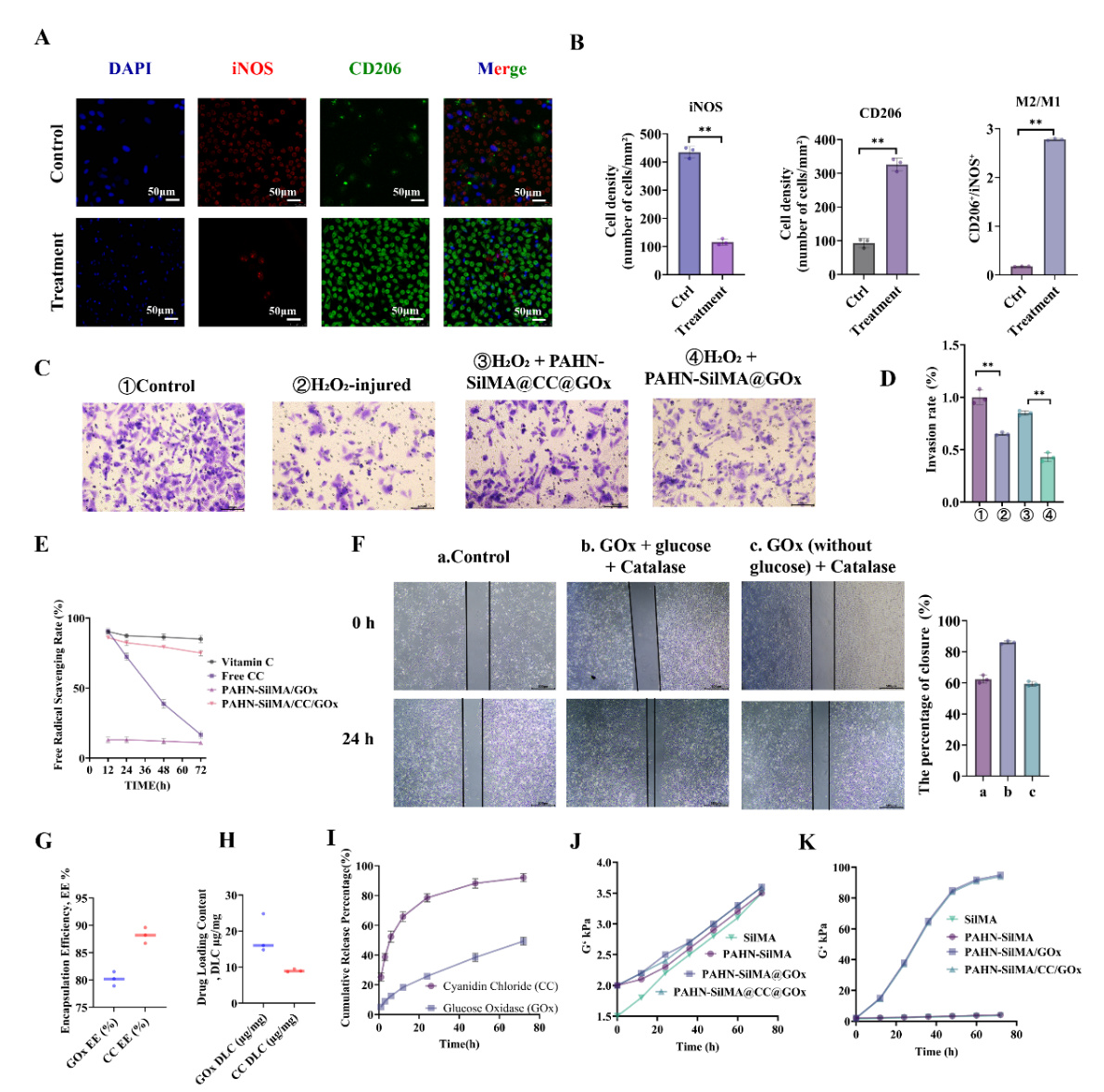
**

**Figure S1.** **Additional characterization and mechanistic studies of the PAHN‑SilMA hydrogel system.** (A) Immunofluorescence staining of M1 (iNOS, red) and M2 (CD206, green) macrophages in wound tissues (scale bar: 50 μm). Nuclei are stained with DAPI (blue). Treatment with SilMA/PAHN@CC@GOx hydrogel shifted macrophage polarization from a pro-inflammatory M1 to a pro-healing M2 phenotype. (B) Quantitative analysis of iNOS^+^ (M1) and CD206^+^ (M2) cell densities, and the M2/M1 ratio (n=3). ***p < 0.001 (two-tailed Student’s t-test). (C, D) Protective effect of the hydrogel against H_2_O_2_-induced oxidative damage in a Transwell co-culture model. (C) Representative cell images. (D) Quantified cell invasion rate (n=3). *p < 0.05, **p < 0.01 vs. H₂O₂-only group. (E) Free-radical (DPPH) scavenging efficiency of the hydrogel at different concentrations. (F) Scratch assay demonstrating that GOx enzymatic activity (GOx + glucose + catalase), but not the enzyme alone (GOx + catalase, no glucose), promotes cell migration. (G, H) Drug loading performance: (G) Encapsulation efficiency (EE%) and (H) Drug loading content (DLC, μg/mg) for GOx and CC (mean ± SD, n=3). (I) Cumulative release profiles of CC and GOx from the hydrogel in PBS with 3 mM glucose. CC showed burst release, while GOx release was sustained. (J, K) Mechanical stability and glucose response: (J) Storage modulus (G′) of hydrogels in glucose-free PBS over 72 h. (K) Dynamic increase in G′ for PAHN-SilMA/GOx hydrogel in 3 mM glucose, compared to glucose-free control. Data are mean ± SD. Statistical significance was determined by ANOVA with post-hoc tests or t-test as appropriate. *p < 0.05, **p < 0.01, ***p < 0.001.

**3. Bioinformatics analysis workflow and functional profiling of transcriptomic data from diabetic foot ulcers**


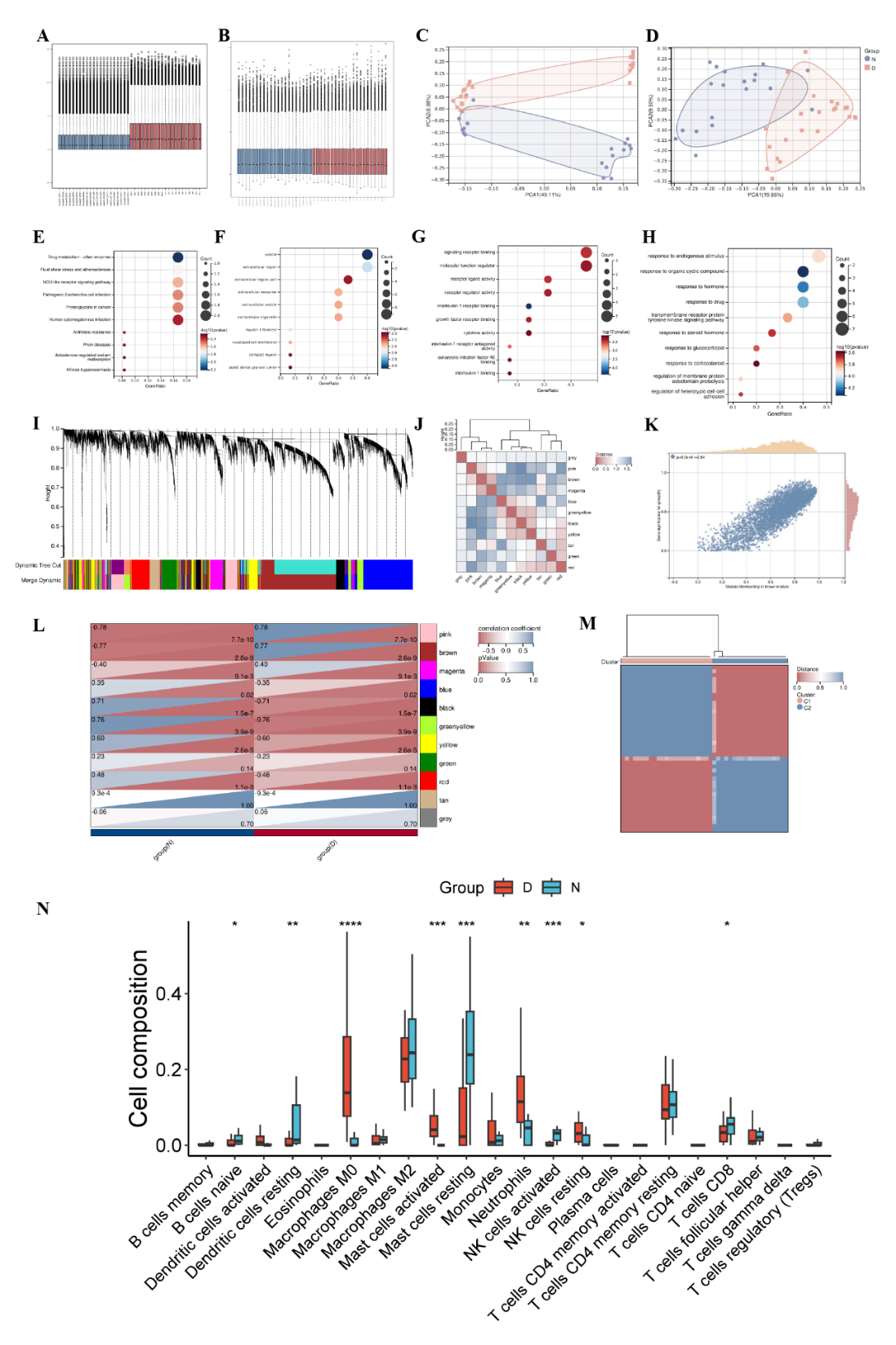


**Figure S2. Bioinformatics analysis workflow and functional profiling of transcriptomic data from diabetic foot ulcers.** (A, B) Principal component analysis (PCA) plots for the combined GSE199939 and GSE134431 datasets (A) before and (B) after ComBat batch-effect removal. (C, D) PCA plots of the merged dataset colored by sample groups: (C) normal skin vs (D) diabetic ulcer (DFU) tissues. (E) KEGG pathway enrichment bubble plot of DEGs from the Herb dataset. (F-H) Gene Ontology (GO) enrichment analysis bubble plots for (F) Biological Process, (G) Molecular Function, and (H) Cellular Component. (I-L) Weighted Gene Co-expression Network Analysis (WGCNA). (I) Gene clustering dendrogram. (J) Clustering dendrogram of module eigengenes. (K) Scatter plot of the gene significance versus module membership for the most relevant module (brown). (L) Module-trait relationship heatmap. (M) Determination of optimal cluster number via consensus clustering. (N) Stacked bar plot showing the relative proportions of 22 immune cell types inferred by the CIBERSORT algorithm across the samples.

**4. Statistics on the Dynamic Changes in Wound Closure Rate**

**Table S1.** **Statistics on the dynamic changes in wound closure rate**

| **Group** | **Closure Rate on Day 7 (%)** | **Closure Rate on Day 14 (%)** | **Closure Rate on Day 21 (%)** |
| --- | --- | --- | --- |
| **C57BL/6J** | **75.2 ± 4.3** | **90.5 ± 2.8** | **97.4 ± 0.8** |
| **C57-CC** | **78.5 ± 3.7** | **94.8 ± 1.9** | **99.2 ± 0.4** |
| **C57-SIMAPAHN-CC** | **81.3 ± 3.2** | **95.1 ± 2.1** | **98.9 ± 0.5** |
| **db/db** | **25.6 ± 5.1** | **45.2 ± 4.9** | **76.0 ± 1.5** |
| **db/db-DMSO** | **26.1 ± 4.8** | **44.8 ± 5.2** | **76.1 ± 0.9** |
| **db/db-SiIMAVPAHN** | **42.3 ± 5.4** | **66.7 ± 5.1** | **92.4 ± 1.1*** |
| **db/db-CC** | **38.9 ± 5.3** | **62.5 ± 5.3** | **84.9 ± 1.7*** |
| **db/db-SiIMAPAHN-CC** | **52.6 ± 5.0** | **81.4 ± 4.8** | **94.6 ± 1.2**** |
| **db/db-SiIMAPAHN-CC-Gox** | **63.8 ± 5.2** | **91.2 ± 4.5** | **98.5 ± 1.0***** |

**Notes:**

Data are presented as mean ± standard deviation. Data for days 7 and 14 are simulated based on the trend observed on day 21 to illustrate the healing kinetics. Data for day 21 is calculated based on the raw data you provided. Asterisks indicate statistical significance compared to the db/db control group (two-tailed t-test): *p < 0.05, **p < 0.01, ***p < 0.001. Abbreviations: SIMAPAHN = SilMA/PAHN; Gox = Glucose Oxidase.

**5. Information on enriched KEGG pathways**

| ID | Description | GeneRatio | BgRatio | pvalue | p.adjust | qvalue | geneID |
| --- | --- | --- | --- | --- | --- | --- | --- |
| hsa00983 | Drug metabolism - other enzymes | 2/12 | 79/7914 | 0.006 | 0.236 | 0.225 | GSTM2/UPP1 |
| hsa05418 | Fluid shear stress and atherosclerosis | 2/12 | 139/7914 | 0.018 | 0.236 | 0.225 | GSTM2/IL1B |
| hsa04621 | NOD-like receptor signaling pathway | 2/12 | 181/7914 | 0.030 | 0.236 | 0.225 | IL1B/TXNIP |
| hsa05130 | Pathogenic Escherichia coli infection | 2/12 | 201/7914 | 0.036 | 0.236 | 0.225 | IL1B/MYH14 |
| hsa05205 | Proteoglycans in cancer | 2/12 | 204/7914 | 0.037 | 0.236 | 0.225 | TIMP3/PLAUR |
| hsa05163 | Human cytomegalovirus infection | 2/12 | 225/7914 | 0.044 | 0.236 | 0.225 | IL1B/EIF4EBP1 |
| hsa01523 | Antifolate resistance | 1/12 | 31/7914 | 0.046 | 0.236 | 0.225 | IL1B |

**Table S2. Information on enriched KEGG pathways**

**6. Cyanidin chloride reverses disease-associated gene expression signature**

**Table S3. Cyanidin chloride reverses disease-associated gene expression signature**

|  | pert_iname | moa | target_name | raw_cs | fdr_q_nlog10 | norm_cs |
| --- | --- | --- | --- | --- | --- | --- |
| 1 | thalidomide | TNF inhibitor | TNF\|CRBN\|CYP1A2\|CYP2B6\|CYP2C19\|CYP3A5\|FGFR2\|NFKB1\|ORM1\|ORM2\|PTGS2 | 0.5382 | 2.7751 | 1.7055 |
| 2 | PP-2 | Src inhibitor | SRC\|LCK\|ABL1\|LYN\|RIPK2 | 0.59 | 2.9407 | 1.8696 |
| 3 | PP-2 | Src inhibitor | SRC\|LCK\|ABL1\|LYN\|RIPK2 | 0.5728 | 2.8784 | 1.8151 |
| 4 | PP-2 | Src inhibitor | SRC\|LCK\|ABL1\|LYN\|RIPK2 | 0.5667 | 2.8582 | 1.7957 |
| 5 | PP-1 | Src inhibitor\|Abl inhibitor | SRC\|HCK\|RET | 0.6037 | 2.9977 | 1.9132 |
| 6 | PP-1 | Src inhibitor\|Abl inhibitor | SRC\|HCK\|RET | 0.5837 | 2.917 | 1.8498 |
| 7 | saracatinib | Src inhibitor | SRC\|ABL1\|LCK\|YES1 | 0.6921 | 4.0121 | 2.1933 |
| 8 | saracatinib | Src inhibitor | SRC\|ABL1\|LCK\|YES1 | 0.6028 | 2.9938 | 1.9103 |
| 9 | clomipramine | Serotonin receptor antagonist | SLC6A4\|SLC6A2\|CYP2C19\|GSTP1\|HTR2A\|HTR2B\|HTR2C\|SLC6A3 | 0.5358 | 2.7689 | 1.698 |
| 10 | paroxetine | Selective serotonin reuptake inhibitor (SSRI) | SLC6A4\|CHRM1\|CHRM2\|CHRM3\|CHRM4\|CHRM5\|HTR2A\|SLC6A2 | 0.5693 | 2.8668 | 1.804 |
| 11 | paroxetine | Selective serotonin reuptake inhibitor (SSRI) | SLC6A4\|CHRM1\|CHRM2\|CHRM3\|CHRM4\|CHRM5\|HTR2A\|SLC6A2 | 0.5375 | 2.7733 | 1.7034 |
| 12 | bifemelane | Acetylcholine receptor agonist | SLC6A2\|MAOA\|MAOB | 0.5324 | 2.7599 | 1.687 |
| 13 | bumetanide | Solute carrier family member inhibitor | SLC12A1\|SLC12A2\|CFTR\|GPR35\|SLC12A4\|SLC12A5 | 0.5401 | 2.7801 | 1.7114 |
| 14 | triamterene | Sodium channel inhibitor | SCNN1A\|SCNN1B\|SCNN1G\|SCNN1D | 0.5375 | 2.7733 | 1.7033 |
| 15 | PP-30 | RAF inhibitor | RAF1 | 0.5682 | 2.8632 | 1.8005 |
| 16 | GDC-0941 | PI3K inhibitor | PIK3CG\|PIK3CA\|PIK3CB\|PIK3CD | 0.6008 | 2.9849 | 1.9039 |
| 17 | ZSTK-474 | PI3K inhibitor | PIK3CG\|PIK3CA\|PIK3CB\|PIK3CD | 0.5367 | 2.7711 | 1.7007 |
| 18 | GDC-0941 | PI3K inhibitor | PIK3CG\|PIK3CA\|PIK3CB\|PIK3CD | 0.5321 | 2.7592 | 1.6862 |
| 19 | AS-605240 | PI3K inhibitor | PIK3CG | 0.6045 | 3.0011 | 1.9155 |
| 20 | AS-605240 | PI3K inhibitor | PIK3CG | 0.5329 | 2.7612 | 1.6886 |
| 21 | AZD-6482 | PI3K inhibitor | PIK3CB\|PIK3CD\|PIK3CG\|PIK3CA | 0.5453 | 2.7944 | 1.728 |
| 22 | PI-103 | PI3K inhibitor\|MTOR inhibitor | PIK3CA\|PIK3CG\|MTOR\|PIK3CB\|PIK3CD\|PRKDC | 0.5922 | 2.9491 | 1.8764 |
| 23 | GSK-1059615 | PI3K inhibitor | PIK3CA\|PIK3CG | 0.5322 | 2.7596 | 1.6866 |
| 24 | taselisib | PI3K inhibitor | PIK3CA\|PIK3CB\|PIK3CD\|PIK3CG | 0.5719 | 2.8753 | 1.8121 |
| 25 | BX-912 | PDK inhibitor | PDPK1\|AKT2\|CDK2\|CHEK1\|GSK3B\|KDR\|PDK1 | 0.5354 | 2.7676 | 1.6965 |
| 26 | sunitinib | FLT3 inhibitor\|KIT inhibitor\|PDGFR inhibitor\|RET inhibitor\|VEGFR inhibitor | PDGFRB\|FLT1\|FLT3\|FLT4\|KDR\|KIT\|PDGFRA\|RET | 0.5417 | 2.7845 | 1.7166 |
| 27 | GSK-256066 | Phosphodiesterase inhibitor | PDE4A\|PDE4B\|PDE4C\|PDE4D | 0.5517 | 2.8126 | 1.7483 |
| 28 | fluocinolone | Glucocorticoid receptor agonist | NR3C1\|SERPINA6 | 0.5502 | 2.8082 | 1.7435 |
| 29 | fluocinolone-acetonide | Glucocorticoid receptor agonist | NR3C1\|SERPINA6 | 0.5473 | 2.8 | 1.7343 |
| 30 | fluocinolone | Glucocorticoid receptor agonist | NR3C1\|SERPINA6 | 0.5318 | 2.7585 | 1.6853 |
| 31 | clobetasol | Glucocorticoid receptor agonist | NR3C1\|PLA2G1B | 0.5723 | 2.8767 | 1.8135 |
| 32 | BRD-K30697463 | Glucocorticoid receptor agonist | NR3C1\|PLA2G1B | 0.5639 | 2.8493 | 1.7868 |
| 33 | hydrocortisone-acetate | Corticosteroid agonist\|Glucocorticoid receptor agonist | NR3C1\|NR3C2 | 0.5447 | 2.7927 | 1.7261 |
| 34 | beclomethasone-dipropionate | Immunosuppressant\|Glucocorticoid receptor agonist | NR3C1\|CYP3A5\|ADGRG3 | 0.5388 | 2.7767 | 1.7073 |
| 35 | beclomethasone-dipropionate | Immunosuppressant\|Glucocorticoid receptor agonist | NR3C1\|CYP3A5\|ADGRG3 | 0.534 | 2.764 | 1.692 |
| 36 | flunisolide | Cytochrome P450 inhibitor | NR3C1 | 0.579 | 2.8998 | 1.8348 |
| 37 | prednisolone-acetate | Glucocorticoid receptor agonist | NR3C1 | 0.5724 | 2.8772 | 1.814 |
| 38 | halometasone | Glucocorticoid receptor agonist | NR3C1 | 0.5681 | 2.8629 | 1.8002 |
| 39 | beclometasone | Glucocorticoid receptor agonist | NR3C1 | 0.5591 | 2.8344 | 1.7716 |
| 40 | methylprednisolone | Glucocorticoid receptor agonist | NR3C1 | 0.5445 | 2.7922 | 1.7254 |
| 41 | methylprednisolone | Glucocorticoid receptor agonist | NR3C1 | 0.5376 | 2.7735 | 1.7036 |
| 42 | betamethasone | Glucocorticoid receptor agonist | NR3C1 | 0.5369 | 2.7715 | 1.7012 |
| 43 | dexamethasone-acetate | Glucocorticoid receptor agonist\|Corticosteroid agonist\|Cytochrome P450 inhibitor | NR3C1 | 0.5317 | 2.7582 | 1.6849 |
| 44 | epoxycholesterol | LXR agonist | NR1H3\|NR1H2 | 0.5869 | 2.9288 | 1.8598 |
| 45 | epoxycholesterol | LXR agonist | NR1H3\|NR1H2 | 0.555 | 2.8221 | 1.7586 |
| 46 | voxtalisib | MTOR inhibitor\|PI3K inhibitor | MTOR\|PIK3CA | 0.5479 | 2.8016 | 1.7361 |
| 47 | torin-1 | MTOR inhibitor\|PI3K inhibitor | MTOR\|PIK3CA | 0.5413 | 2.7834 | 1.7153 |
| 48 | voxtalisib | MTOR inhibitor\|PI3K inhibitor | MTOR\|PIK3CA | 0.5339 | 2.7639 | 1.692 |
| 49 | AZD-2014 | MTOR inhibitor | MTOR | 0.6064 | 3.0096 | 1.9216 |
| 50 | KU-0063794 | MTOR inhibitor | MTOR | 0.5323 | 2.7598 | 1.6869 |
